# Die Situation pflegender Angehöriger im erwerbsfähigen Alter in der COVID-19-Pandemie – Ergebnisse einer Onlinebefragung in Deutschland

**DOI:** 10.1007/s00103-023-03659-7

**Published:** 2023-02-07

**Authors:** Henrik Wiegelmann, Moritz Hess, Dominik Domhoff, Franziska Heinze, Annika Schmidt, Kathrin Seibert, Claudia Stolle, Benedikt Preuß, Heinz Rothgang, Karin Wolf-Ostermann

**Affiliations:** 1 Institut für Public Health und Pflegeforschung, Abteilung Pflegewissenschaftliche Versorgungsforschung, Universität Bremen, Grazer Straße 4, 28359 Bremen, Deutschland; 2 SOCIUM Forschungszentrum Ungleichheit und Sozialpolitik, Universität Bremen, Bremen, Deutschland; 3 Zentrum für Pflegeforschung und Beratung, Hochschule Bremen, Bremen, Deutschland; 4 Hochschule Niederrhein, Niederrhein, Deutschland

**Keywords:** Pflegende Angehörige, Lebensqualität, Pflegebelastung, COVID-19-Pandemie, Häusliche Pflege, Informal caregivers, Quality of life, Burden of care, COVID-19 pandemic, Home-based care

## Abstract

**Hintergrund:**

Die Folgen der COVID-19-Pandemie haben verschiedene Personengruppen vor große Herausforderungen gestellt; eine dieser Gruppen sind pflegende Angehörige. Die vorliegende Studie untersucht, welche Veränderungen die Pandemie für pflegende Angehörige mit sich gebracht hat und in welchem Ausmaß sich Lebensqualität und Pflegebelastung subgruppenspezifisch verändert haben.

**Methode:**

Die Datenerhebung erfolgte im Sommer 2020 in einer Querschnittsstudie mit pflegenden Angehörigen im erwerbsfähigen Alter (*N* = 1143). Neben soziodemografischen Daten wurden Angaben zu Versorgungssituation, Vereinbarkeit von Pflege und Beruf sowie Belastung und Lebensqualität in einer Onlinebefragung erhoben. Versorgungssituation und Vereinbarkeit von Pflege und Beruf wurden deskriptiv analysiert. Für die Analysen der Veränderung der Lebensqualität und der Belastung wurden logistische Regressionsmodelle verwendet.

**Ergebnisse:**

Die Versorgungssituation hat sich für viele Befragte (54,7 %) während der Pandemie geändert und ist zeitlich aufwendiger geworden. Für 70,8 % ist die Vereinbarkeit von Pflege und Beruf schwieriger geworden. Mit dem Pandemiemanagement der eigenen Arbeitgeber:innen zeigt sich die Mehrheit zufrieden (65,9 %). Die Lebensqualität hat ab- und die Belastung zugenommen, besonders deutlich für jüngere Pflegende, Frauen und Pflegende von Personen mit hohem Pflegebedarf.

**Diskussion:**

Die Ergebnisse weisen darauf hin, dass sich die Lebenssituationen pflegender Angehöriger während der COVID-19-Pandemie verschlechtert haben. Entscheidungsträger:innen sollten dies anerkennen und besonders betroffene Subgruppen pflegender Angehöriger unterstützen. Zukünftig ist es wichtig, die informelle häusliche Pflege ebenso wie Versorgungssettings der professionellen (Langzeit‑)Pflege in gesundheits- und sozialpolitische Pandemiekonzepte einzubeziehen.

## Abstract (en)

**Background:**

The consequences of the COVID-19 pandemic have posed major challenges to different groups. One of these are informal caregivers. This study investigates the changes the pandemic has caused for informal caregivers and the extent to which quality of life and burden of care have changed for specific subgroups.

**Methods:**

Data for this cross-sectional study was gathered in the summer of 2020 in a convenient sample of informal caregivers (< 67 years of age, *N* = 1143). In addition to sociodemographic data, information on the care situation, compatibility of care and work, as well as stress and quality of life was collected in an online survey. The analysis of care situations and compatibility of care and work is done descriptively. Logistic regression models are used for a subgroup analysis of quality of life and care burden.

**Results:**

The care situation has changed for 54.7% of participants and has become more time consuming. For 70.8% of respondents, the COVID-19 pandemic has made it even more difficult to balance care-giving and work. However, most respondents were satisfied with their employers’ pandemic management (65.9%). A sharp decline in the quality of life and an increase in the burden of care for informal caregivers was ascertained. Both developments are stronger for young and female caregivers and for those caring for people with a greater need of support.

**Discussion:**

The results indicate that living situations worsened for a substantial proportion of informal caregivers during the COVID-19 pandemic. Policymakers should recognize additional challenges that informal caregivers have faced since the outbreak of the COVID-19 pandemic and how they vary by subgroups. It is important to include home-based informal care as well as other care settings in future pandemic concepts.

## Hintergrund

In vielen Ländern rund um den Globus hat die steigende Lebenserwartung dazu geführt, dass die Zahl der älteren Menschen, absolut und relativ gesehen, zunimmt [1]. Dies hat zu einem raschen Anstieg der Zahl der Pflegebedürftigen geführt, da die Wahrscheinlichkeit, eine chronische Krankheit zu entwickeln oder Einschränkungen im täglichen Leben zu erfahren, mit dem Alter zunimmt [2].

In Deutschland haben im Dezember 2021 ca. 4,9 Mio. Menschen Pflegeversicherungsleistungen aus der gesetzlichen Pflegeversicherung oder der privaten Pflichtversicherung bezogen [3]. Rund 80 % der Pflegebedürftigen werden innerhalb der häuslichen Umgebung versorgt [4]. Mehr als 90 % der häuslich versorgten Personen verfügen über eine oder mehrere Hauptpflegepersonen [5], die auch bei Einschaltung eines Pflegedienstes den größten Teil der Versorgungsleistungen erbringen [6]. Ungefähr 70 % der pflegenden Angehörigen sind weiblich, Repräsentativbefragungen der letzten Jahre veranschaulichen jedoch einen wachsenden Anteil männlicher pflegender Angehöriger, der 2018 bei ca. 30 % lag [5]. Die Pflege wird von Partner:innen (34 %), Kindern (32 %), Schwiegerkindern (7 %), Eltern (15 %), anderen Verwandten (5 %) und Freund:innen oder Nachbar:innen (7 %) übernommen [5]. In gut einem Drittel der häuslichen Pflegearrangements übernimmt eine Person die Pflege allein, 28 % werden von 2 Personen betreut und in 31 % der Fälle sind 3 oder mehr Personen in die Versorgung involviert [5].

Bereits vor der COVID-19-Pandemie war die Situation in der häuslichen Pflege Gegenstand einer Vielzahl von wissenschaftlichen Untersuchungen. Häufig wurde dabei belegt, dass die Versorgung einer pflegebedürftigen Person für viele pflegende Angehörige – trotz der Existenz von Entlastungsmöglichkeiten – mit teilweise starken Einschränkungen ihrer Lebenssituation einhergeht. So wurden erhöhte Belastungen zum Beispiel bei der physischen und psychischen Gesundheit dokumentiert und Verschlechterungen hinsichtlich der Lebensqualität und der Möglichkeiten der gesellschaftlichen Teilhabe nachgewiesen [4, 7]. Neben den gesundheitlichen und psychischen Belastungen können auch finanzielle Risiken, die sich zum Beispiel aus Verdienstausfall ergeben, pflegende Angehörige belasten [3, 6, 8].

Während der COVID-19-Pandemie hat sich der Kontext der Pflege grundlegend verändert [9]. Erstens sind pflegebedürftige Menschen nach einer Infektion einem hohen Risiko ausgesetzt, schwer zu erkranken [10]. Dies erzeugt Ängste und Unsicherheit bei Pflegenden und Pflegebedürftigen. Die Vermeidung von Infektionen ist daher von größter Bedeutung und häufig werden Maßnahmen der physischen Distanzierung zu pflegbedürftigen Personen umgesetzt, was sich negativ auf das Wohlbefinden und die soziale Gesundheit von älteren Menschen mit Pflegebedarf auswirken kann [11]. Schon früh in der Pandemie haben Studien gezeigt, dass insbesondere die Quarantänemaßnahmen negative psychische und gesundheitliche Folgen für die Betroffenen haben können [12, 13].

Zweitens wird die Unterstützung in der eigenen Häuslichkeit durch professionelle Pflegepersonen aufgrund gesetzlicher Vorschriften für Anbieter:innen, selbst auferlegter Beschränkungen oder aus Angst vor Ansteckung durch die professionellen Pflegepersonen möglicherweise reduziert [14–16]. Dies kann zu einer erhöhten Belastung für pflegende Angehörige führen, da sie die weggefallene Unterstützung kompensieren müssen. Untersuchungen aus dem Vereinigten Königreich zeigen, dass sich die COVID-19-Pandemie auf pflegende Angehörige ausgewirkt hat, die von erhöhter Unsicherheit und Kontrollverlust sowie einer schlechteren Lebensqualität berichten [14, 15]. Ergebnisse aus Spanien und England zeigen ein erhöhtes Stressniveau bei pflegenden Angehörigen [17]. Erste Ergebnisse aus Deutschland zeigen, dass die formelle und informelle soziale Unterstützung während der ersten Welle der Pandemie erheblich abnahm [18]. Für ein Drittel der Befragten hat die Pandemie zudem ihre Situation als pflegende Angehörige verschlechtert und ebenso viele sind besorgt, dass sie unter den gegebenen Umständen nicht in der Lage sind, eine angemessene Pflege zu leisten [18]. Für Deutschland wird berichtet, dass soziale Isolation und ein erhöhter Pflegeaufwand als belastend empfunden werden [19]. Auch leiden während der Pandemie insbesondere pflegende Angehörige mit einer hohen Pflegebelastung (höhere Pflegegrade und Pflege von Menschen mit Demenz; aus den Pflegeaufgaben resultierende Belastung; [20]). Diese Ergebnisse deuten auf einen negativen Einfluss der COVID-19-Pandemie auf pflegende Angehörige in Bezug auf Lebensqualität und Pflegebelastung hin.

Ziel dieser Studie ist es, erlebte Veränderungen der Lebenssituation pflegender Angehöriger während der COVID-19-Pandemie sichtbar zu machen sowie Möglichkeiten und Grenzen der Durchführung häuslicher Pflege unter erschwerten Pandemiebedingungen zu dokumentieren. Neben der Versorgungssituation und der Vereinbarkeit von Pflege und Beruf stehen daher auch die Lebensqualität pflegender Angehöriger und die Pflegebelastung im Fokus der Studie.

Die Studie adressiert folgende Forschungsfragen:

- Wie wirkte sich die COVID-19-Pandemie auf die Versorgungssituation in häuslichen Pflegearrangements aus?
- Wie wirkte sich die COVID-19-Pandemie auf die Vereinbarkeit von Pflege und Beruf aus und wie bewerten die Befragten das Pandemiemanagement ihrer Arbeitgeber:innen?
- Wie wirkte sich die COVID-19-Pandemie auf die Lebensqualität und die Pflegebelastung aus und welche Subgruppen sind davon besonders betroffen?

## Methode

### Studiendesign und Durchführung der Studie

Die vorliegende Studie ist eine Querschnittsstudie auf Basis einer Gelegenheitsstichprobe, die im Sommer 2020 (08.06.–12.08.) nach der ersten COVID-19-Welle im Rahmen einer Kooperation der Universität Bremen, der DAK-Gesundheit und dem Selbstvertretungsverein „wir pflegen e. V.“ durchgeführt wurde. Die Datenerhebung erfolgte online mittels EFS-Survey, einer Onlineumfragesoftware. Der Fragebogen wurde in Abstimmung mit Vertreter:innen des Vereins „wir pflegen e. V.“ partizipativ erarbeitet. Eine detaillierte Beschreibung der Studie findet sich im Projektbericht [21].

Zielgruppe der Befragung waren Personen im erwerbsfähigen Alter bis 67 Jahre, die der Definition einer Pflegeperson gemäß § 19 Elftes Buch Sozialgesetzbuch (SGB XI) entsprechen.^1^ Die auf diese Weise identifizierten pflegenden Angehörigen wurden mit einem postalischen Anschreiben durch die DAK-Gesundheit auf die Befragung aufmerksam gemacht. Das Datenschutzkonzept wurde mit der Datenschutzbeauftragten der Universität Bremen abgestimmt. Alle Befragten wurden vor Studienbeginn schriftlich über das Datenschutzkonzept informiert.

### Datenaufbereitung und Datenauswertung

Die Befragungsdaten wurden vor der Auswertung auf Plausibilität geprüft. Antworten außerhalb des gültigen Wertebereichs wurden von der Auswertung ausgeschlossen. Bei fehlenden Daten wurde die befragte Person jeweils nur für das betreffende Item aus der Auswertung ausgeschlossen. Die für die Prozentberechnungen verwendeten Stichprobengrößen der berücksichtigten gültigen Fälle unterscheidet sich daher von Item zu Item. Alle statistischen Auswertungen wurden mit Stata und IBM SPSS Statistics durchgeführt.

### Messinstrumente

Um *allgemeine Veränderungen der Versorgungssituation* zu identifizieren, wurden die Befragten gebeten, 2 Einzelfragen zu beantworten. Zum einen wurde erfragt, ob sich seit Ausbruch der COVID-19-Pandemie etwas an der Versorgungssituation bei der zu pflegenden Person verändert hat. Eine weitere Frage zielte darauf ab, die Art der Veränderung hinsichtlich einer Reihe vorgegebener formeller Versorgungsangebote zu identifizieren. Die Befragten konnten angeben, dass a) es keine Veränderungen der Inanspruchnahme gegeben hat, b) sie Angebote und Dienstleistungen wegen der Pandemie nicht mehr in Anspruch nehmen wollen oder c) Angebote und Dienstleistungen wegen der Pandemie nicht mehr angeboten werden. Fälle, für die einzelne Versorgungsangebote nicht zutrafen, wurden für die hier vorgenommenen deskriptiven Analysen itembezogen ausgeschlossen, was die Anzahl gültiger Angaben reduzierte.

Auch zur *Vereinbarkeit von Pflege und Beruf* wurden den Befragten 2 allgemeine Einzelfragen gestellt. Zuerst wurde erfragt, ob seit Ausbruch der COVID-19-Pandemie die Vereinbarkeit von Pflege und Beruf weniger Probleme, gleich viele Probleme oder mehr Probleme bereitet. In der zweiten Frage ging es um das Pandemiemanagement der Arbeitgeber:innen und darum, ob die pflegenden Angehörigen damit zufrieden, teils zufrieden/teils unzufrieden oder nicht zufrieden sind. Die zugehörigen deskriptiven Analysen beziehen sich auf erwerbstätige pflegende Angehörige.

Um die *Veränderungen in der Lebensqualität* der pflegenden Angehörigen zu messen, wurden 2 Einzelfragen zur Lebensqualität vor und während der COVID-19-Pandemie verwendet. Die Befragten konnten auf einer 5‑stufigen Likert-Skala antworten (sehr schlecht, schlecht, durchschnittlich, gut, sehr gut). Aus den beiden Variablen wurde eine dritte Variable mit den Ausprägungen 1 = Verschlechterung der Lebensqualität, 2 = keine Veränderung der Lebensqualität/Verbesserung der Lebensqualität erstellt (eine dritte Kategorie entfiel für den vorliegenden Datensatz, da eine geringere Belastung von niemandem berichtet wurde).

Analog zur Messung der Lebensqualität wurden Veränderungen der *Pflegebelastung* aus 2 Fragen zur Pflegebelastung vor und während der COVID-19-Pandemie abgeleitet, die die Befragten auf einer 5‑stufigen Likert-Skala beantworten konnten (sehr belastet, ziemlich belastet, etwas belastet, kaum belastet, überhaupt nicht belastet). Auch hier wurde eine Variable mit 2 Kategorien konstruiert: 1 = mehr Belastung durch die Pflege, 2 = keine Veränderung der Belastung durch die Pflege. Detaillierte Darstellungen zu den Variablen Lebensqualität und Pflegebelastung finden sich im Ergebnisteil in Tab. 3. Eine detaillierte Beschreibung der Messinstrumente findet sich im Projektbericht [21].

### Analysemethoden

Für die Analysen haben wir deskriptive Darstellungen zur allgemeinen Übersicht der Ergebnisse mit Regressionstechniken kombiniert, die eingesetzt wurden, um subgruppenspezifische Veränderungen der Lebensqualität und der Belastung durch Pflege zu untersuchen. Die Variablen „Lebensqualität“ und „Pflegebelastung“ dienten als abhängige Variablen in den statistischen Modellen. Da es sich um dichotome abhängige Variablen handelt, wurden multivariate logistische Regressionsmodelle verwendet und Odds Ratios berechnet. Um zu untersuchen, ob sich die potenziellen Auswirkungen der COVID-19-Pandemie auf die Lebensqualität und Pflegebelastung der Pflegenden zwischen den Untergruppen der pflegenden Angehörigen unterscheiden, wurden die folgenden erklärenden Variablen in die Analysemodelle aufgenommen: Geschlecht (männlich, weiblich), Altersgruppen (< 50, 50–60, > 60), Bildungsniveau (nicht-tertiär, tertiär), Beschäftigungsstatus (erwerbstätig, nicht erwerbstätig), Leben im selben Haushalt (ja, nein) und Pflegegrad (PG; PG 1 und 2, 3, 4, 5) der zu pflegenden Person. Da der Anteil der Personen mit PG 1 unter einem Prozent lag, wurden PG 1 und 2 zusammengefasst.

## Ergebnisse

### Beschreibung der Stichprobe

Insgesamt nahmen 1143 pflegende Angehörige an der Onlinebefragung teil (Rücklaufquote: 4,7 %). Tab. 1 fasst zentrale soziodemografische Kennziffern der Stichprobe zusammen. 86 % der Befragten sind Frauen und etwa ein Viertel verfügt über einen Hochschulabschluss. Ungefähr ein Drittel der Befragten ist jünger als 50 Jahre. Etwas weniger als die Hälfte ist erwerbstätig und 4 von 5 Befragten leben mit der gepflegten Person zusammen. Ein Viertel der zu pflegenden Personen hat den PG 1 oder 2, die größte Teilgruppe (34,3 %) hat den PG 3. Auf den Pflegegrad 4 entfallen 22,9 % und auf den PG 5 entfallen 17,5 %.

**Table 1:** 

Variablen	Anteil in % und Anzahl
*Geschlecht*	
Weiblich	86,0 (*n* = 980)
Männlich	14,0 (*n* = 159)
*Altersgruppen*	
< 50 Jahre	34,9 (*n* = 397)
50–60 Jahre	41,5 (*n* = 471)
> 60 Jahre	23,6 (*n* = 268)
*Bildung*	
Tertiärer Bildungsabschluss (Hochschule)	26,3 (*n* = 291)
Nicht-tertiärer Bildungsabschluss	73,7 (*n* = 817)
*Beschäftigungsstatus*	
Formell beschäftigt (Arbeit, Ausbildung, Schule)	49,2 (*n* = 557)
Nicht formell beschäftigt	50,8 (*n* = 574)
*Wohnsituation Angehörige und gepflegte Person*	
Zusammenlebend	80,5 (*n* = 893)
Nicht zusammenlebend	19,5 (*n* = 217)
*Pflegegrade (der gepflegten Person)*	
1 und 2	25,4 (*n* = 276)
3	34,3 (*n* = 373)
4	22,9 (*n* = 249)
5	17,5 (*n* = 172)

### Veränderungen der Versorgungssituation

Insgesamt geben 54,7 % der befragten pflegenden Angehörigen an, dass die COVID-19-Pandemie ihre Versorgungssituation verändert hat (nicht tabellarisch dargestellt). Frauen (57,8 %) berichten häufiger eine Veränderung als männliche pflegende Angehörige (45,5 %). Dies trifft auch auf jüngere pflegende Angehörige unter 50 Jahren (63,4 %) im Vergleich zur Gruppe zwischen 50–60 Jahren (52,9 %) und der Gruppe über 60 Jahren (51,3 %) zu. Pflegende Angehörige mit tertiärer Bildung (66,7 %) geben häufiger an, Veränderungen erlebt zu haben, als Angehörige ohne tertiäre Bildung (52,2 %). Befragte ohne eine weitere formelle Beschäftigung (Beruf, Ausbildung, Schule) geben häufiger an, von Veränderungen betroffen zu sein, als beschäftigte pflegende Angehörige (61,5 % vs. 50,3 %). Liegt ein höherer PG vor (4 und 5), dann berichten Befragte häufiger Veränderungen als bei niedrigeren PG (PG 1 und 2: 45,4 %; PG 3: 53,4 %). Hinsichtlich der Wohnsituation (zusammenlebend/nicht zusammenlebend) zeigen sich zu vernachlässigende Differenzen (56,3 % vs. 55,1 %). Weitere Informationen zu Veränderungen der Versorgungssituation können dem Projektbericht entnommen werden [21].

**Figure 1:**
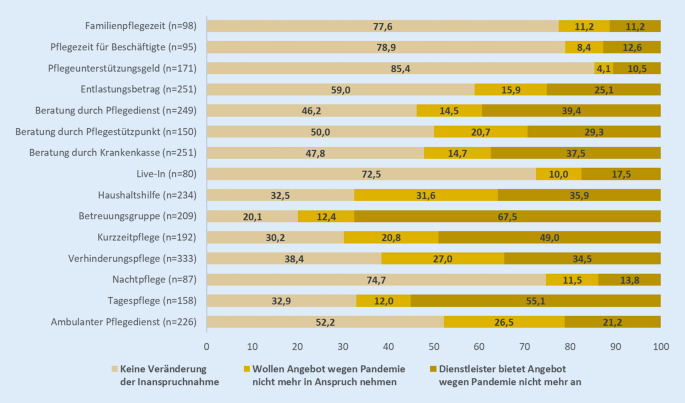

Mit Blick auf die Art der Veränderung der Versorgungssituation zeigt sich eine *rückläufige Inanspruchnahme* für alle formellen Angebote, bei einer insgesamt gering ausfallenden Inanspruchnahme (Abb. 1, Mehrfachnennungen möglich). Am stärksten rückläufig ist die Inanspruchnahme bei der Verhinderungspflege (z. B. Urlaubsvertretung, Vertretung im Krankheitsfall), der Beratung durch Pflegedienste, den häuslichen Besuchsdiensten und den Betreuungsgruppen. Dabei ist die Ursache für den Rückgang der Nutzung sowohl darauf zurückzuführen, dass Dienstleistende das Angebot aufgrund der COVID-19-Pandemie nicht mehr anbieten, als auch darauf, dass die Pflegehaushalte das Angebot wegen der Pandemie nicht mehr in Anspruch nehmen. Allerdings ist der Wegfall des Angebots bei fast allen Diensten der entscheidende Grund. Lediglich bei der Nutzung der ambulanten Pflegedienste ist die Reduktion der Nachfrage quantitativ bedeutsamer als eine Reduktion des Angebots.

Die 3 Angebote mit der höchsten Kontinuität (keine Veränderung trotz Pandemie) sind die Familienpflegezeit, Pflegezeit für Beschäftigte sowie das Pflegeunterstützungsgeld. Dies sind Leistungen, die keinen direkten physischen Kontakt mit sich bringen und damit hinsichtlich einer möglichen Infektion mit SARS-CoV‑2 kein Risiko darstellen. Zu den 3 Angeboten, die von den befragten Angehörigen am häufigsten nicht mehr in Anspruch genommen werden wollten, zählen Haushaltshilfen, Verhinderungspflege sowie ambulante Pflegedienste. Die 3 Angebote, welche vonseiten der Dienstleistenden am häufigsten eingestellt wurden, sind Betreuungsgruppen, Tagespflege sowie die Kurzzeitpflege. Sowohl die Angebote, die von den Angehörigen nicht mehr in Anspruch genommen werden wollten, als auch die vonseiten der Dienstleistenden eingestellten Angebote sind Leistungen, die für gewöhnlich mit einem hohen Ausmaß an physischem Kontakt einhergehen und damit als potenzielle Risikoträger für eine SARS-CoV-2-Infektion gelten können.

Eine rückläufige Nutzung ambulanter Pflegedienste erhöht – unter sonst gleichen Bedingungen – den Bedarf an informeller Pflege. Dementsprechend gibt etwas mehr als die Hälfte (54,9 %) der Befragten an, dass sich der tägliche Zeitaufwand für die Pflege in der COVID-19-Pandemie erhöht hat (nicht tabellarisch dargestellt). Frauen berichten dies häufiger als Männer (57,4 % vs. 39,1 %), jüngere pflegende Angehörige häufiger als Angehörige mittleren (50–60 Jahre) Alters (54 %) und höheren (über 60 Jahre) Alters (43,6 %). Befragte mit tertiärer Bildung (57,6 %) geben häufiger an, einen Mehraufwand zu haben, als Befragte ohne tertiäre Bildung (53,9 %). Wenn gepflegte und pflegende Person zusammenleben, wird vermehrt ein höherer Aufwand berichtet, als wenn sie nicht zusammenleben (55,7 % vs. 50,7 %). Mit ansteigendem PG steigt durchgehend auch der berichtete Mehraufwand (PG 1 und 2: 45,9 %; PG 3: 53,7 %; PG 4: 60,6 %; PG 5: 63,6 %). Hinsichtlich der Berufstätigkeit (beschäftigt vs. nicht-beschäftigt) sind keine Unterschiede zu verzeichnen; für beide Gruppen lässt sich eine Erhöhung des Zeitaufwands feststellen. Dieser erhöhte Zeitaufwand dürfte insbesondere auf die veränderten Rahmenbedingungen zurückgehen, wie unter anderem geringere soziale Unterstützung, erhöhter Aufwand für die Bewältigung des Alltags mit Pflegebedürftigen, geringere Unterstützung durch versorgende Institutionen, mehr Personen im Haushalt unter Quarantänebedingungen, oder auch auf veränderte Anforderungen bei der Erwerbsarbeit. Weitere Informationen hierzu können dem Projektbericht entnommen werden [21].

### Vereinbarkeit von Pflege und Beruf sowie Zufriedenheit mit den Arbeitgeber:innen

Ein Großteil der befragten erwerbstätigen pflegenden Angehörigen (70,8 %) gibt an, während der ersten Welle der Pandemie mehr Probleme mit der Vereinbarkeit von Pflege und Beruf gehabt zu haben, als dies vor der Pandemie der Fall war (Tab. 2). Für ein Viertel (26,4 %) der erwerbstätigen Angehörigen haben sich die Vereinbarkeitsprobleme nicht verändert. Mit Blick auf soziodemografische Merkmale zeigen sich Unterschiede nach bestimmten Subgruppen. Vermehrte Probleme werden vor allem von Frauen berichtet, von jüngeren pflegenden Angehörigen, von Angehörigen mit tertiärer Bildung sowie von den mit der gepflegten Person zusammenlebenden Angehörigen. Darüber hinaus steigt mit dem PG der Anteil derjenigen Angehörigen, die angeben, während der Pandemie mehr Probleme zu haben als zuvor (Tab. 2).

**Table 2:** 

**Veränderung bei der Vereinbarkeit von Pflege und Beruf (in %)**			
–	Weniger Probleme als zuvor	Gleich viele Probleme wie zuvor	Mehr Probleme als zuvor
*Insgesamt*	2,7 (*n* = 14)	26,4 (*n* = 137)	70,8 (*n* = 367)
*Geschlecht*			
Weiblich	2,8	24,2	73,0
Männlich	1,9	46,3	51,9
*Alter*			
Unter 50 Jahre	1,6	21,1	77,4
50 bis 60 Jahre	3,6	28,5	68,0
Älter als 60 Jahre	2,9	35,7	61,4
*Bildung*			
Tertiäre Bildung	4,7	21,5	73,8
Nicht-tertiäre Bildung	2,0	28,6	69,4
*Beschäftigung (Beruf, Ausbildung, Schule)*			
Ja	2,7	26,9	70,4
Nein	–	–	–
*Wohnsituation*			
Zusammenlebend	2,8	24,4	72,8
Nicht zusammenlebend	2,6	33,6	63,8
*Pflegegrad*			
1 und 2	3,5	35,9	60,6
3	1,8	27,4	70,8
4	2,0	20,4	77,6
5	4,5	14,6	80,9
**Zufriedenheit mit dem Pandemiemanagement der Arbeitgeber:innen (in %)**			
–	Zufrieden	Teils, teils	Nicht zufrieden
*Insgesamt*	65,9 (*n* = 323)	20,0 (*n* = 98)	14,1 (*n* = 69)
*Geschlecht*			
Weiblich	65,7	20,5	13,9
Männlich	67,3	16,3	16,3
*Alter*			
Unter 50 Jahre	65,6	18,0	16,4
50 bis 60 Jahre	65,0	21,5	13,5
Älter als 60 Jahre	70,0	20,0	10,0
*Bildung*			
Tertiäre Bildung	71,9	10,4	17,8
Nicht-tertiäre Bildung	62,8	23,9	13,3
*Beschäftigung (Beruf, Ausbildung, Schule)*			
Ja	66,0	20,2	13,8
Nein	–	–	–
*Wohnsituation*			
Zusammenlebend	67,4	19,3	13,4
Nicht zusammenlebend	63,1	20,7	16,2
*Pflegegrad*			
1 und 2	71,7	14,5	13,8
3	60,8	22,9	16,3
4	65,6	21,5	12,9
5	65,5	23,8	10,7

Parallel zu der allgemeinen Zunahme der wahrgenommenen Vereinbarkeitsprobleme, berichtet die Mehrheit der erwerbstätigen Angehörigen (65,9 %), dass sie mit dem Pandemiemanagement ihrer Arbeitgeber:innen zufrieden waren, und nur 14,1 % der Befragten äußerten Unzufriedenheit mit ihren Arbeitgeber:innen. Auch wenn die Pandemie mutmaßlich bei den meisten Befragten in hohem Ausmaß berufliche Veränderungen bewirkt hat [22], führt das nur in geringerem Maße zu Konflikten zwischen Arbeitgeber:innen und den berufstätigen pflegenden Angehörigen. Auch hier lassen sich Unterschiede bezüglich soziodemografischer Merkmale identifizieren (Tab. 2). Geringfügige Unterschiede zeigen sich beim Geschlecht; Männer sind etwas häufiger mit dem Pandemiemanagement ihrer Arbeitgeber:innen zufrieden. Zufriedener sind tendenziell auch ältere Angehörige (älter als 60 Jahre), Angehörige mit tertiärer Bildung, Angehörige, die mit der gepflegten Person zusammenleben, und Angehörige, bei denen die gepflegte Person einen PG 1 oder 2 hat.

### Veränderungen der Lebensqualität und der Pflegebelastung

Tab. 3 zeigt die Verteilung der abhängigen Variablen Lebensqualität und Pflegebelastung vor und während der COVID-19-Pandemie. Insgesamt hat sich die Lebensqualität für die Hälfte der befragten pflegenden Angehörigen (49,9 %) nach eigenen Angaben verschlechtert und für einen noch etwas höheren Anteil (55 %) hat die Pflegebelastung seit Beginn der COVID-19-Pandemie zugenommen. Das heißt, sie haben vor der Pandemie eine höhere Lebensqualität und eine niedrigere Pflegebelastung angegeben.

**Table 3:** 

Lebensqualität			Belastung durch Pflege		
	Vor COVID-19-Pandemie (%)	Während COVID-19-Pandemie (%)		Vor COVID-19-Pandemie	Während COVID-19-Pandemie
Sehr schlecht	0,8	8,2	Sehr belastet	6,1	37,8
Schlecht	5,9	22,1	Ziemlich belastet	34,8	34,5
Mittel	33,6	37,0	Etwas belastet	42,5	21,2
Gut	46,1	26,7	Kaum belastet	13,6	5,0
Sehr gut	13,6	6,0	Überhaupt nicht belastet	3,0	1,6
Verringerung der Lebensqualität (%)	49,9 (*n* = 514)		Gestiegene Belastung durch Pflege (%)	55,0 (*n* = 567)	

### Multivariate Analyse der Lebensqualität und Pflegebelastung

Tab. 4 zeigt die Ergebnisse der logistischen Regressionen bei denen Lebensqualität und Pflegebelastung als abhängige Variablen und Geschlecht, Alter, Bildung, Erwerbstätigkeit, Zusammenleben mit Pflegebedürftigen und Pflegegrad als unabhängige Variablen einbezogen wurden. Die Wahrscheinlichkeit, eine Verschlechterung der Lebensqualität und eine Zunahme der Pflegebelastung wahrgenommen zu haben, ist für Frauen, Personen mit Pflegebedürftigen höherer PG und Jüngere höher als für Männer, Personen mit Pflegebedürftigen niedrigerer PG und Ältere. Es wurden keine signifikanten Zusammenhänge für Bildung, Beschäftigung oder Zusammenleben mit dem pflegebedürftigen Angehörigen gefunden.

**Table 4:** 

	Verschlechterung der Lebensqualität		Höhere Belastung durch Pflege	
	Odds Ratio	95 %-Konfidenzintervall	Odds Ratio	95 %-Konfidenzintervall
*Geschlecht (Ref.: männlich)*				
Weiblich	1,95***	1,33–2,85	1,77**	1,21–2,59
*Altersgruppen (Ref.: <* *50 Jahre)*				
50–60 Jahre	0,67*	0,50–0,91	0,55***	0,40–0,76
> 60 Jahre	0,66*	0,47–0,93	0,33***	0,22–0,47
*Bildung (Ref.: keine Hochschule)*				
Hochschule	0,82	0,61–1,11	0,99	0,73–1,35
*Beschäftigungsstatus (Ref.: nicht beschäftigt)*				
Beschäftigt	0,79	0,60–1,03	0,90	0,68–1,19
*Zusammenlebend (Ref.: nein)*				
Ja	1,21	0,86–1,69	0,87	0,62–1,22
*Pflegegrad (Ref.: 1 und 2)*				
3	1,49*	1,06–2,09	1,24	0,88–1,74
4	1,83**	1,25–2,67	1,43	0,96–2,10
5	1,69*	1,13–2,53	2,03**	1,33–3,10
*N*	*966*		*966*	
*Pseudo‑R*^*2*^	*0,03*		*0,06*	

* *p* < 0,05, ** *p* < 0,01, *** *p* < 0,001

## Diskussion

Die Ergebnisse zeigen, dass die allgemeine Versorgungssituation sich für mehr als die Hälfte der befragten pflegenden Angehörigen (59 %) während der COVID-19-Pandemie geändert hat. Für 71 % der Befragten ist die Vereinbarkeit von Pflege und Beruf mit mehr Problemen verbunden. Gleichzeitig zeigt sich jedoch die Mehrheit mit dem Pandemiemanagement der eigenen Arbeitgeber:innen zufrieden (60 %). Die Befragten gehen somit davon aus, dass die erschwerte Situation den objektiven Umständen und nicht dem individuellen Fehlverhalten von Arbeitgeber:innen geschuldet ist. Die Wahrscheinlichkeit, eine Verschlechterung der Lebensqualität und eine Zunahme der subjektiven Pflegebelastung wahrgenommen zu haben, findet sich für jüngere Pflegende, Frauen und Angehörige von Gepflegten mit hohem Pflegebedarf.

Die Ergebnisse der vorliegenden Studie stimmen in verschiedener Hinsicht mit weiteren Befragungen zur Situation pflegender Angehöriger in Deutschland während der COVID-19-Pandemie überein. Zum Beispiel werden Rückgänge in der Inanspruchnahme von formellen Unterstützungsangeboten und/oder -leistungen beziehungsweise eine Verschlechterung des Zugangs zu Gesundheits- und Sozialdiensten auch in anderen Studien berichtet [18, 23, 24]. Ebenfalls zeigt sich über verschiedene Studien hinweg, dass die Vereinbarkeit von Pflege und Beruf für die Personengruppe pflegender Angehöriger im Durchschnitt schwieriger geworden ist [18, 23, 24]. Weiterführende Analysen zeigen darüber hinaus, dass pflegende Angehörige von Menschen mit Demenz häufiger von Schwierigkeiten mit der Vereinbarkeit betroffen sind und dass die Sorge um den eigenen Arbeitsplatz besonders in niedrigen Einkommensgruppen vorhanden ist [25]. Was das Pandemiemanagement der eigenen Arbeitgeber:innen betrifft, bestätigt eine andere Untersuchung die relativ hohen Zufriedenheitswerte [18]. Eine weitere groß angelegte Befragung im Auftrag des Sozialverbands VdK Deutschland kommt hier jedoch auf einen deutlich niedrigeren Wert [23].

Die hier vorliegende Studie zeigt darüber hinaus eine Abnahme der Lebensqualität der Pflegenden und eine Zunahme der subjektiven Pflegebelastung für die Hälfte der Befragten. Dieses Ergebnis steht im Einklang mit früheren Untersuchungen, die einen Rückgang der Lebensqualität von Pflegenden im Vereinigten Königreich festgestellt haben [15]. Für Deutschland konnten diese Effekte bestätigt werden [26]. Weitere in Deutschland durchgeführte Studien bestätigen ebenfalls die negativen Entwicklungen im Bereich der psychosozialen Gesundheit pflegender Angehöriger, zum Beispiel mit Blick auf die subjektive Belastung, Ängste und Sorgen sowie das Einsamkeitsempfinden [18, 23, 24, 27]. Es bleibt abzuwarten, ob es sich hierbei um nachhaltige Veränderungen handelt, die über die Pandemie hinaus anhalten. Erste Analysen legen nahe, dass es sich hierbei auch um temporäre Entwicklungen handeln könnte und Erholungseffekte im Pandemieverlauf stattgefunden haben [27]. Hier bedarf es sicherlich zukünftig weiterer Forschungsaktivitäten.

Die vorliegende Studie leistet einen Beitrag zur Forschung über die Auswirkungen der COVID-19-Pandemie auf die Lebenssituationen pflegender Angehöriger, trägt zum Forschungsstand bei, indem sie negative Dynamiken subgruppenspezifisch analysiert und dabei zeigt, dass die schlechtere Lebensqualität und die erhöhte subjektive Pflegebelastung während der Pandemie besonders für weibliche und junge Pflegende sowie für solche mit schwer beeinträchtigten Pflegebedürftigen von Bedeutung sind. Bestimmte Subgruppen pflegender Angehöriger scheinen Belastungen demnach stärker wahrzunehmen. Dies steht auch in Einklang mit Ergebnissen der CORONA-HEALTH-App-Studie in Deutschland, die in Bezug auf verschiedene Aspekte von Lebensqualität insbesondere für Frauen und jüngere Personen niedrigere Werte im Vergleich zu Werten aus der Zeit vor der COVID-19-Pandemie aufzeigt [28]. Ein Befund, der darauf hinweist, dass eine adäquate Zielgruppenorientierung bei der Ausgestaltung von Unterstützungsangeboten zur Förderung der mentalen Gesundheit berücksichtigt werden sollte [29].

Ferner stimmen die Ergebnisse mit früheren Untersuchungen überein, die zeigen, dass Frauen während der COVID-19-Pandemie am stärksten benachteiligt sind [26, 30, 31]. Obwohl jüngere Pflegepersonen während der Pandemie mit zusätzlichen Herausforderungen wie Kinderbetreuung und Berufstätigkeit zu kämpfen haben könnten [32], wurde kein signifikanter Zusammenhang mit der Erwerbstätigkeit festgestellt. Die Ergebnisse zu Personen mit intensiveren Betreuungspflichten stimmen mit den Ergebnissen einer weiteren Befragung von pflegenden Angehörigen aus Deutschland überein [18]. Die Pflegearrangements von Menschen mit höheren PG umfassen häufig auch formelle Pflege, was bedeutet, dass die Belastung für pflegende Angehörige zunimmt, wenn diese Unterstützung ausläuft oder beendet wird.

### Limitationen

Bei der Interpretation der Ergebnisse sind folgende Einschränkungen zu beachten: Die erhobenen Daten beruhen auf Einschätzungen der Befragten und sind daher keine objektiven Bezugsgrößen. Pflegende, die nicht erwerbstätig sind, nicht bei der DAK versichert sind und nicht über einen Internetzugang verfügen, wurden nicht in die Gruppe der Befragten eingeschlossen und der Rücklauf war gering. Es handelt sich bei der Stichprobe also nicht um eine Zufallsstichprobe, die Repräsentativität gewährleistet, sondern um eine Gelegenheitsstichprobe, bei der aufgrund der Selbstselektivität der Befragten Rückschlüsse auf die Eigenschaften der Grundgesamtheit aller pflegenden Angehörigen im erwerbsfähigen Alter nur in eingeschränktem Maße möglich und zulässig sind. Daher können die Ergebnisse nicht auf die gesamte Gruppe pflegender Angehöriger übertragen werden. Daten zur vergleichenden Einschätzung der Lebensqualität bei nicht informell Pflegenden im erwerbstätigen Alter fehlen. Zudem handelt es sich bei den in der Analyse verwendeten Daten um Querschnittsdaten mit retrospektiven Fragen und nicht um echte Längsschnitte. Verzerrungen der Einschätzungen aufgrund verzerrter Erinnerungen („recall bias“) sind daher nicht auszuschließen. Es ist weiterhin zu beachten, dass wir aufgrund geringer Fallzahlen die zu Pflegenden der Pflegegrade 1 und 2 zusammenfassend analysiert haben. Die Datenerhebung wurde im Anschluss an die erste COVID-19-Welle durchgeführt und die Interpretationen sind daher auf diesen Zeitraum beschränkt.

### Schlussfolgerungen

Die vorliegenden Ergebnisse stärken die Forderung nach einer differenzierte(re)n Analyse von Bedarfssituationen in der häuslichen Pflege sowie darauf abgestimmten zielgruppenspezifischen Unterstützungsangeboten, die auf vorhandenen finanziellen, sozialen und psychologischen Ressourcen aufbauend die Resilienz pflegender Angehöriger stärken [33]. Neuere Studien verweisen darauf, dass Resilienzförderung bei pflegenden Angehörigen auf 3 Ebenen stattfinden sollte: der Angehörigenebene (z. B. Selbstwirksamkeitserwartung, Coping-Strategien), der dyadischen Ebene (z. B. Beziehungsqualität, Reziprozität) sowie der Angehörigen-Umwelt-Interaktion (z. B. soziale Unterstützung; [33]). Dem hinzuzufügen wäre noch eine strukturelle Ebene, auf der eine vorbeugende Pflege- und Sozialpolitik zur Stärkung häuslicher Versorgungsarrangements realisiert wird [34].

Künftige Forschungsarbeiten sollten daher diese Ebenen stärker einbeziehen und auch im Zeitverlauf analysieren, um so potenzielle Einflussmechanismen auf Lebensqualität und Belastung der pflegenden Angehörigen – auch über die COVID-19-Pandemie hinaus – zu identifizieren. Und nicht zuletzt sollten die Erkenntnisse zu Belastungen pflegender Angehöriger stärker in der Öffentlichkeit wahrgenommen und diskutiert werden, um so Grundlagen für verbesserte Rahmenbedingungen zu schaffen und politische Entscheidungsträger:innen zu sensibilisieren.

## Footnotes

1 Gemäß § 19 Elftes Buch Sozialgesetzbuch (SGB XI) werden pflegende Angehörige als Pflegepersonen anerkannt, wenn folgende Kriterien erfüllt sind: a) Es wird nicht erwerbsmäßig eine pflegebedürftige Person (nach § 14) in der eigenen Häuslichkeit gepflegt, b) die Pflege umfasst mindestens 10 h pro Woche bei mindestens einer pflegebedürftigen Person, verteilt auf regelmäßig mindestens 2 Tage in der Woche. Die befragten Personen erhalten Leistungen zur sozialen Sicherung nach § 44 SGB XI.
